# Chronic myeloid leukemia progenitor cells require autophagy when leaving hypoxia-induced quiescence

**DOI:** 10.18632/oncotarget.18904

**Published:** 2017-06-30

**Authors:** Angela Ianniciello, Pierre-Yves Dumas, Claire Drullion, Amélie Guitart, Arnaud Villacreces, Yan Peytour, Jean Chevaleyre, Philippe Brunet de la Grange, Isabelle Vigon, Vanessa Desplat, Muriel Priault, Persio Dello Sbarba, Zoran Ivanovic, François-Xavier Mahon, Jean-Max Pasquet

**Affiliations:** ^1^ Cellules Souches Hématopoïétiques Normales et Leucémiques, INSERM U1035 BMGIC, Université de Bordeaux, 33076 Bordeaux Cedex, France; ^2^ UMR CNRS 5095, I.B.G.C, Université de Bordeaux, 33077 Bordeaux Cedex, France; ^3^ Department of Experimental and Clinical Biomedical Sciences, Università degli Studi di Firenze, 50134 Firenze, Italia; ^4^ INSERM U1218, Institut Bergonié, 33076 Bordeaux, France; ^5^ Etablissement Français du Sang Aquitaine-Limousin, 33075 Bordeaux, France

**Keywords:** chronic myeloid leukemia, autophagy, stem cell

## Abstract

Albeit tyrosine kinase inhibitors anti-Abl used in Chronic Myeloid Leukemia (CML) block the deregulated activity of the Bcr-Abl tyrosine kinase and induce remission in 90% of patients, they do not eradicate immature hematopoietic compartments of leukemic stem cells. To elucidate if autophagy is important for stem cell survival and/or proliferation, we used culture in low oxygen concentration (0.1% O_2_ for 7 days) followed back by non-restricted O_2_ supply (normoxic culture) to mimic stem cell proliferation and commitment. Knockdown of *Atg7* expression, a key player in autophagy, in K562 cell line inhibited autophagy compared to control cells. Upon 7 days at 0.1% O_2_ both K562 and K562 shATG7 cells stopped to proliferate and a similar amount of viable cells remained. Back to non-restricted O_2_ supply K562 cells proliferate whereas K562 shATG7 cells exhibited strong apoptosis. Using immunomagnetic sorted normal and CML CD34^+^ cells, we inhibited the autophagic process by lentiviral infection expressing shATG7 or using a Vps34 inhibitor. Both, normal and CML CD34^+^ cells either competent or deficient for autophagy stopped to proliferate in hypoxia. Surprisingly, while normal CD34^+^ cells proliferate back to non restricted O_2_ supply, the CML CD34^+^ cells deficient for autophagy failed to proliferate. All together, these results suggest that autophagy is required for CML CD34^+^ commitment while it is dispensable for normal CD34 cells.

## INTRODUCTION

Chronic myeloid leukemia (CML) is a clonal malignant hematopoietic disorder characterized by the presence of a t(9;22)(q34;q11) reciprocal translocation [1, 2]. The BCR-ABL hybrid gene, the molecular hallmark of CML [3] encodes an oncogenic fusion protein harboring a deregulated tyrosine kinase activity that is responsible for leukemogenesis *in vitro* and *in vivo* [4, 5]. Development of ABL1 tyrosine kinase inhibitors (TKI) in the past decade provided the proof of concept that targeted therapies are an attractive strategy in CML. TKI imatinib is now the front-line therapy of CML in chronic phase, and competes with ATP for binding to the Abl kinase domain. Because imatinib resistance has been a well-recognized problem, particularly in the advanced phase of the disease, new TKI have been developed. Nilotinib and dasatinib, the second-generation TKI, have been developed to override primary and secondary resistance [6, 7].

Hematopoietic stem cells (HSC) perpetuate a continuous stream of differentiated blood cells. In CML, the transformed HSC called leukemic stem cells (LSC) initiate and sustain the disease. Currently, the remaining questions are how to avoid TKI therapy for all life and how to eradicate the disease. Most of LSC reside in hematopoietic niches with peculiar biophysical conditions that preserve them. Indeed, the concentrations of oxygen in the organism are very different from the atmospheric concentration and varies according to the tissues [8]. The hematopoietic niche is characterized by low oxygen concentrations ranging from 4 to 0.1% [9–11]. TKI used in CML patient treatment are not able to eliminate these CSL and treatment abortion is followed by 60% of relapse [12–14]. Several studies reported that targeting other pathways in combination with TKI treatment could be efficient enough to target LSC. Among these interesting pathways, the inhibition of autophagy has been reported to be deleterious on leukemia and on the LSC reservoir [15]. This interesting result has led to propose preclinical and clinical trials in CML using autophagy inhibitors. To date, results are disappointing while all previous studies were very encouraging. Indeed, autophagy is involved in degradation of long life components or organelles. It is triggered by stress conditions like nutriment starvation and used a complex machinery involving ATG proteins [16, 17].

We aimed at investigating if autophagy is required when stem cells leave the hematopoietic niche and if this requirement is similar between normal and CML cells. To answer this, we employed a strategy in which cells are placed for 7 days at low concentration of oxygen allowing slow cycling (LC1) and then replaced at atmospheric oxygen concentration leading to proliferation (LC2) [18].

## RESULTS

### Inhibition of autophagy did not alter viable cell number and level of apoptosis in CML cell culture incubated at low O_2_ concentration

**Figure 1:**
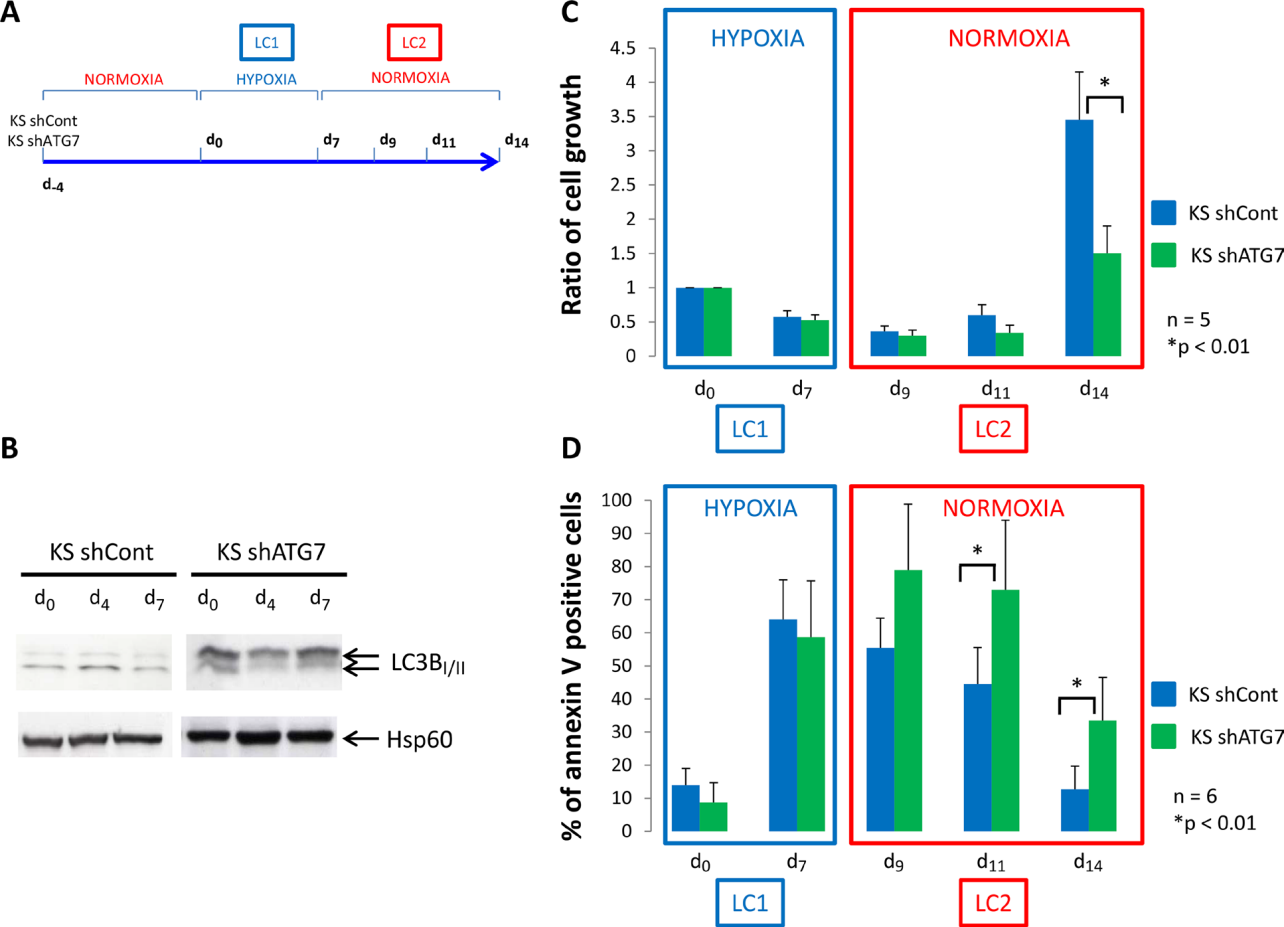
Low oxygen concentration decrease proliferation of K562 cells (**A**) K562 cells expressing a shRNA against luciferase (KS shCont) or a shRNA against ATG7 (K shATG7) were cultured at low O_2_ concentration (hypoxia 0.1% O_2_) for 7 days (LC1). Upon 7 days, cells were replaced at atmospheric O_2_ concentration and grown until day 14 (LC2). (**B**) At day 0, 4 and 7, samples were proceed to analyze conversion of LC3B-I in LC3B-II by western-blot. (**C**) and (**D**) At indicated time, aliquot were analysed for cell count by trypan blue exclusion assay and apoptosis by flow cytometry using annexin V-APC labelling. Results are from at least 5 experiments. Significance between autophagy competent and deficient cells was quantitated using Wilcoxon test and show by an asterisk when *p <* 0.01.

We used the K562 CML cell line in which a control shRNA (KS shCont) or a shRNA against the protein ATG7 (KS shATG7) were expressed through virus infection. Inhibition of ATG7 expression was confirmed by western blotting and the consequent inhibition of autophagy in K562 shATG7 was verified by detecting the conversion of microtubule-associated light chain 3B-I in LC3B-II by western-blotting (Figure 1B). According to the procedure described by Giuntoli *et al.* [18] (Figure 1A), the two cell lines were cultured at 0.1% O_2_ (hypoxia) for 7 days (LC1). Compared to day 0, both cell lines underwent numerical decrease over incubation and significant increase of apoptosis at day 7 (Figure 1C and 1D, LC1).

### Inhibition of autophagy reduce numerical expansion and enhance apoptosis of CML cells following transfer to growth-permissive cultures in atmosphere O_2_ concentration

Back to atmosphere O_2_ concentration (Figure 1C, LC2), culture repopulation by either cell line exhibited a long lag phase. At day 14, KS shCont cells repopulated cultures significantly more efficiently than KS shATG7 cells. KS shATG7 cells exhibited significantly higher levels of apoptosis than control cells (Figure 1D, LC2). Thus, blocking autophagy in the K562 CML cell line strongly impaired proliferation and viability in normoxic LC2 of cells rescued from hypoxic LC1.

### CML CD34^+^ progenitor cells stop cycling in hypoxia

**Figure 2:**
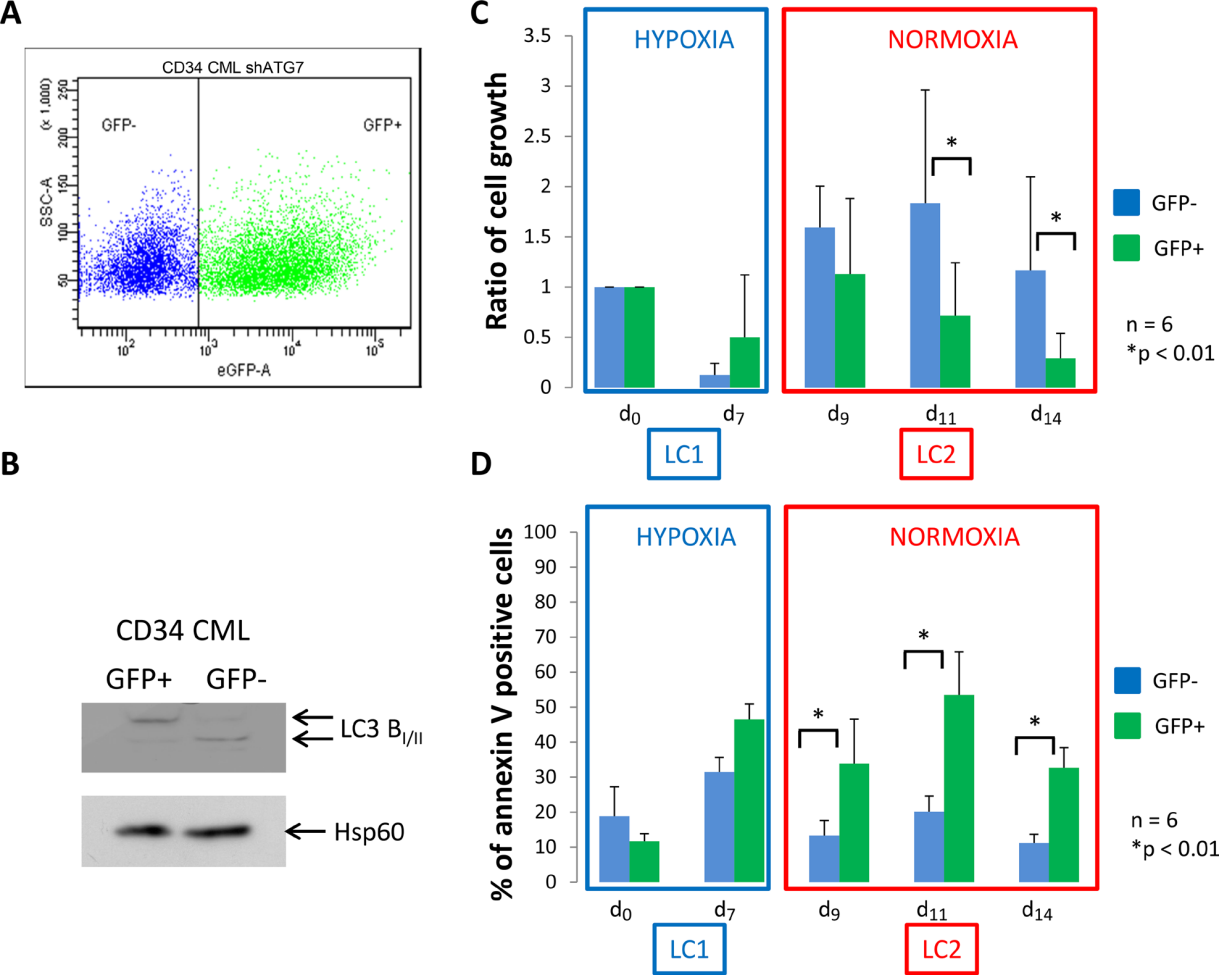
CML CD34^+^ cells deficient for autophagy have impaired proliferation and show apoptosis back to atmospheric O_2_ concentration (**A**) CML CD34^+^ cells were transduced with a shRNA against ATG7 and analyzed by flow cytometry to determine the percent of GFP^-^ and GFP^+^ population. (**B**) Upon cell sorting, GFP^-^ and GFP^+^ cells were lyzed and autophagy operating mechanism was check by detecting the conversion of microtubule associated light chain 3B-I in LC3B-II. CD34^+^ cells were cultured at low O_2_ concentration (0.1% O_2_) for 7 days (LC1). Upon 7 days, cells were replaced at atmospheric O_2_ concentration and grown until day 14 (LC2). (**C**) and (**D**) At indicated time, aliquot were analysed for cell count by trypan blue exclusion assay and apoptosis by flow cytometry using annexin V-APC labelling on the two population GFP^–^ and GFP^+^. Results are from at least 6 experiments. Significance between autophagy competent and deficient cells was quantitated using Wilcoxon test and show by an asterisk when *p <* 0.01.

We next explored if the requirement of autophagy of CML cells is also required for primary CML CD34^+^ cells harvested *ex vivo*. We used lentiviral infection to inhibit expression of ATG7 in immunopurified CD34^+^ CML cells. The plasmid used benefits from a GFP reporter gene allowing to gate cells expressing GFP to select cells expressing shATG7. Four days after the infection, flow cytometry, performed in 6 separate experiments, revealed a GFP+ cell population between 40 and 60% (Figure 2A). To confirm the efficiency of shATG7 expression, GFP+ and GFP- cells were sorted and used for the detection of the conversion of microtubule-associated light chain 3B-I in LC3B-II by western blotting (Figure 2B). GFP^-^ and GFP^+^ cells were placed at low O_2_ concentration for 7 days (Figure 2C and 2D, LC1) and then transferred to growth-permissive cultures incubated in atmosphere O_2_ concentration (Figure 2C and 2D, LC2). Upon 7 days of hypoxia, a large majority of CD34^+^ CML cells, was low cycling, as determined via the detection of the proliferation marker Ki-67 (Supplementary Figure 1A, LC1). Viable cell number and level of apoptosis for CML CD34^+^ cells (Figure 2C and 2D, LC1) behaved like in the case of K562 cells (Figure 1C and 1D, LC1). In atmosphere O_2_ concentration, CD34^+^ CML cells exhibited a kinetics of LC2 repopulation more prompt (no lag phase) than that of K562 cells (Figure 2C vs Figure 1C, LC2). In LC2, GFP^+^ cells underwent a significantly lower expansion and higher apoptosis levels than GFP^-^ cells (Figure 2C and 2D, LC2). To avoid artefactual apoptosis linked to virus infection and ShRNA expression we performed similar experiments using control ShRNA which show no effect on apoptosis (Supplementary Figure 1B). These results emphasized those obtained for K562 shATG7 cells. In GFP^+^ and GPP^-^ CD34^+^ CML cells, expression of BCR/Abl protein was suppressed in hypoxia and re-expressed following transfer to atmosphere O2 concentration (Supplementary Figure 1C), as already reported [18, 19]. Because autophagy appeared requiring for proliferation of CML cells following transfer to atmosphere O_2_ concentration, we tried to confirm this finding by colony assay. Cells were seeded in methylcellulose at days 0, 7 (LC1) and 14 (LC2) in 3 separate experiments and the colonies counted on four fields after 10 days of incubation. The results obtained indicated that the generation of colony-forming cells was progressively reduced during cell population expansion in LC2 and that the impairment of autophagy significantly enhanced this reduction (Supplementary Figure 2).

### Inhibition of autophagy in normal CD34^+^ cells neither change quiescence nor proliferation and viability back to atmosphere O_2_ concentration

**Figure 3:**
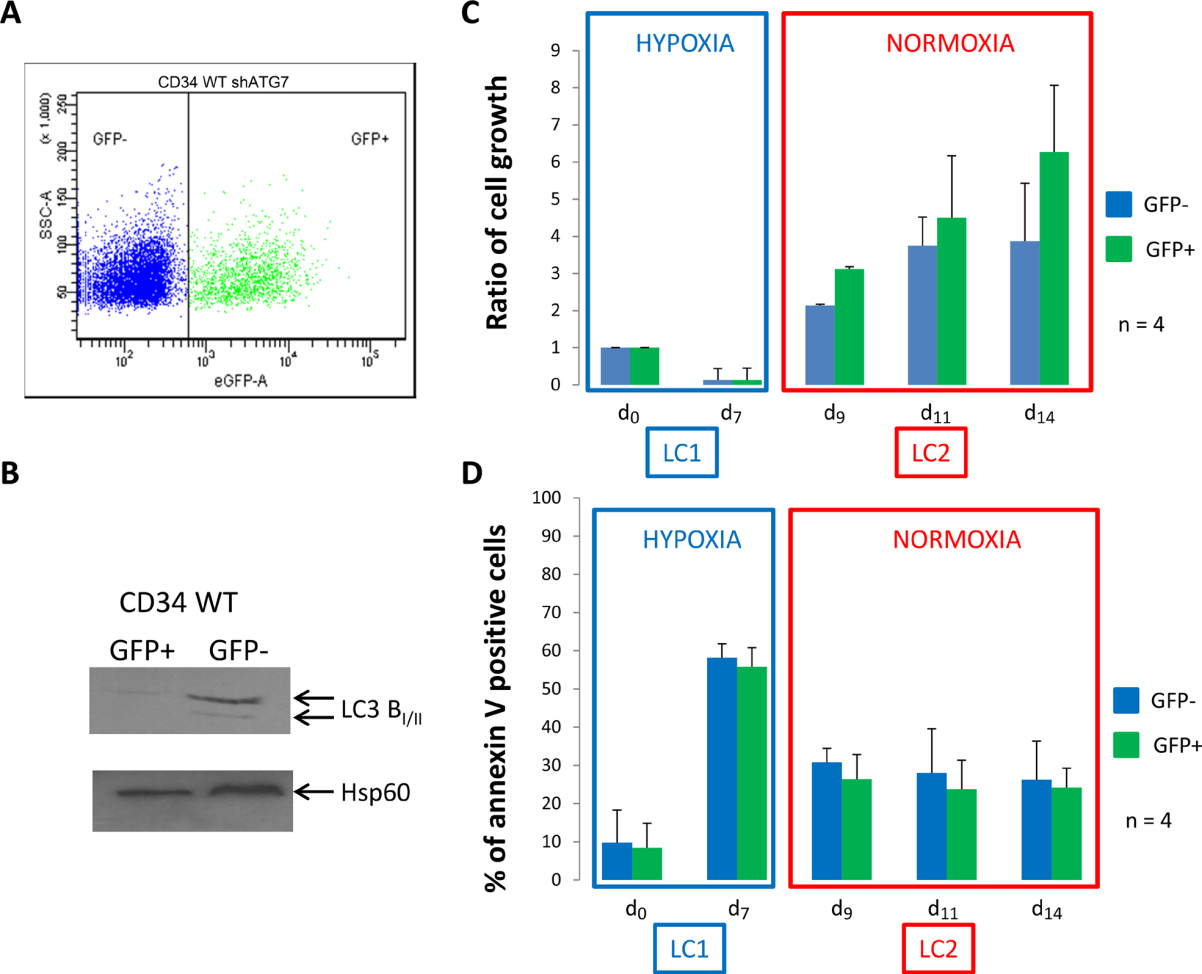
Normal CD34^+^ progenitor cells proliferate and show low apoptosis back to atmospheric O_2_ concentration (**A**) Normal CD34^+^ cells were transduced with a shRNA against ATG7 and analyzed by flow cytometry to determine the percent of GFP^-^ and GFP^+^ population. (**B**) Upon cell sorting, GFP^-^ and GFP^+^ cells were lyzed and autophagy operating mechanism was check by detecting the conversion of microtubule associated light chain 3B-I in LC3B-II. CD34^+^ cells were cultured at low O_2_ concentration (0.1% O_2_) for 7 days (LC1). Upon 7 days, cells were replaced at atmospheric O_2_ concentration and grown for seven more days (LC2). (**C** and **D**) At indicated time, aliquot were analyzed for cell count by trypan blue exclusion assay and apoptosis by flow cytometry using annexin V-APC labelling on the two population GFP^-^ and GFP^+^. Results are from at least 4 experiments. Significance between autophagy competent and deficient cells was quantitated using Wilcoxon test.

Because both *in vitro* and *ex vivo* results suggested that autophagy is required for the full maintenance of LC2 repopulation ability of CML cells incubated in hypoxia, we asked whether it also plays a critical role in normal CD34^+^ cells. Normal CD34^+^ cells were purified from leukoreduction filters as described Peytour *et al* [20]. Four days after infection, normal CD34^+^ cells were analyzed for GFP expression and between 35 and 50% of GFP^+^ cells were detected in 3 independent experiments (Figure 3A). To confirm the efficiency of shATG7, GFP^-^ and GFP^+^ cells were sorted and used for detection by western blotting of the conversion of microtubule-associated light chain 3B-I in LC3B-II (Figure 3B). In addition, inhibition of autophagy was also checked by incubating GFP- and GFP+ cells in minimal medium HBSS and conversion of LC3 was detected by western-blot (Supplementary Figure 3A). Cells were then processed like in the experiments relative to CML cells (Figures 1 and 2). Like CD34^+^ CML cells, normal CD34^+^ cells incubated at low O_2_ concentration for 7 days promptly repopulated normoxic LC2 with no lag phase (Figure 3C, LC2). In contrast to CML cells, normal cells did not exhibit any significant difference between GFP^+^ and GFP^-^ cells (Figure 3D, LC2). These results were emphasized by colony formation assay (Supplementary Figure 3B). This different behaviour is confirmed by pharmacological inhibition of autophagy using a Vps34 inhibitor in WT and CML CD34+ cells [21]. As shown in Supplementary Figure 3C, only CML CD34+ cells underwent apoptosis when Vps34 is blocked emphasizing autophagy requirement for CML commitment.

## DISCUSSION

HSC immaturity and commitment has been linked to an oxygen gradient in bone marrow [9, 22]. Self-renewal and multipotency are key properties of HSC, allowing to sustain life-long blood cell production. In addition to many canonical pathways controlling self-renewal, autophagy has been reported to be critical for blood cell production, as deletion of ATG7 depleted the HSC compartment [23]. However, the same study reports that ATG7 requirement is not observed when foetal liver HSC have been used to reconstitute hematopoiesis. In CML, autophagy protects leukemic cells as it decreases the efficiency of imatinib, so the combination of autophagy inhibition with imatinib enhances CML cell death [15]. In addition, it was reported that primary CML cells, including LSC, are killed when autophagy is inhibited [15, 24, 25]. However, to eradicate LSC, it is necessary to deplete hematopoietic niches from leukemic quiescent cells which are able to commit and proliferate. By using low O_2_ concentration, we were able to reduce cycling enough on both cell lines and CD34^+^ cells. In our hands, 0.1% O_2_ during 7 days inhibits proliferation of a huge proportions of either cell lines or CD34^+^ cells, which is commonly described [26]. Placing hematopoietic cells at low O_2_ concentration decrease metabolism which shift from oxydation to glycolysis. At this stage autophagy do not seems to be required or critical.

When cells are back to atmospheric O_2_ concentration, an oxidative burst occurred. All mechanisms participating to the cell cycle, DNA replication and expression of a large number of proteins involved in cell replication, require to refill all the reserves, and autophagy may be crucial at this step for CML cells. In addition, autophagy can afford protection from oxidative stress in CML cells [15, 27]. However, proliferation of normal CD34^+^ cells is similar for autophagy-competent or -deficient cells. This result suggests that autophagy is dispensable or stress may be overcame by other mechanisms in normal cells. Indeed, autophagy is also controlling the number of mitochondria (mitophagy) and in ATG7 deficient CD34^+^ CML cells the accumulation of damaged organelles may kill the cell. In fact, the metabolic adaptation to the atmospheric O_2_ concentration of normal CD34^+^ cells may still compensate for increasing request by an enhanced energy production, while in CD34^+^ CML cells, autophagy may be required to avoid damages [23]. We and other observed that Bcr-Abl protein expression decreased in hypoxia both in CML cell line or CD34^+^ CML progenitors. The decrease of protein translation in hypoxia is a well known cell response linked to quiescence even not yet fully understood [28–30]. However, for CD34^+^ CML cells autophagy is required when leukemic cells repopulate and reexpressed normal protein level as seen for Bcr-Abl. It will be interesting to check if supplying some metabolites may correct this defect or in an opposite way if starvation of one metabolite is deleterious. As previously reported in acute leukemia, addiction to a metabolite like glutamine may be partially circumvented by autophagy when leukemic cells proliferate [31].

Indeed, CD34^+^ CML cells leaving low O_2_ environment may require strong energy adaptation to be able to repopulate. It is well known that hypoxia regulates mTor, itself regulatory negatively autophagy [32, 33]. What we observed could be an indirect consequence of hypoxia. At 0.1% of O_2_, both normal and leukemic cells cycle slowly and reduce metabolism intensity. They consume a lot of glucose and its concentration decreases enough inducing autophagy through AMPK and mTor-induced phosphorylation of ULK1 [34, 35]. In autophagy-deficient CD34^+^ CML cells, this may occur similarly without inducing autophagy. Back to non-restricted atmospheric O_2_ supply these cells encounter the most difficulties to repopulate. In contrast, in normal CD34^+^ cells this needs may not be required or may be overcame.

Indeed, autophagy requirement when LSC commits may well explain why the inhibition of autophagy in CML patients does not bring a real benefit since this affect only cells that will engage. One possibility to target the CML LSC will be thus to mobilize stem cells in combination with the inhibition of autophagy. HSC seems to be not addicted to autophagy when they engage. This will be a benefit for HSC but deleterious to LSC. Pharmacological autophagy inhibitors recently reported could be a very important way.

In conclusion, tracking autophagy deserves to pay attention but should be considered in combination to stem cell mobilizing agent to be able to target different steps of commitment and stem cell population in CML and then applied to other leukemia.

## MATERIALS AND METHODS

### Reagents

RPMI 1640 medium, fetal calf serum (FCS), phosphate buffered saline (PBS), were from Invitrogen. Trypan blue and the antibody against LC3 were from Sigma (St Quentin Fallavier, France). The following antibodies : ATG7 was from Cell Signalling (Danvers, USA), and Hsp60 was from Santa Cruz (Bergheimer, Germany). Annexin-V-APC was from Beckman coulter (Villepinte, France). Ki-67 antibody, control isotype and anti-Abl were purchased from BD (Becton Dickinson, France). PIK-III and SBI-0206965 were purchased from Selleck chemicals (Houston, USA).

### Cell lines

The human Bcr-Abl positive K562 cell line used in this study was from the *American Type Culture Collection (*ATCC). Cells were maintained in RPMI 1640 medium supplemented with 10% FCS, 2 mM L-glutamine, 100 U/mL penicillin and 0.1 mg/mL streptomycin at 37°C in a humidified atmosphere containing 5 % CO_2_. Aliquots were taken at 24 h intervals for assessment of cell viability by Trypan blue exclusion. The shATG7 K562 cell line was generated as previously described [36].

### CD34 cell isolation

All CML patient samples correspond to diagnosis when patients were in chronic phase with Ph chromosome and BCR-ABL-positive CML. Informed consent was obtained in accordance with the Declaration of Helsinki from all patients by the biological resource center (BRC N° EN-CRB-042). Retrospectively, all patients were responding to TKI. Mononuclear cells were isolated from bone marrow or blood by Ficol gradient. CD34^+^cells were purified using human CD34^+^ cell selection kit (Miltenyi Biotech, Germany) and purity was analyzed by flow cytometry using phycoerythrin-conjugated anti-CD34 antibody (Becton Dickinson, France). Purified CD34^+^ positive cells were frozen in liquid nitrogen until use. For normal CD34^+^ cell, leukoreduction filters were provided by the Blood French institute of Bordeaux and flushed according to the procedure described by Peytour et al [37]. Cytokines and growth factors were from PeproTech (Neuilly sur Seine, France).

### Culture at low or atmospheric oxygen concentration

As reported by Giuntoli *et al*, cells are placed at day zero (d0) at low oxygen concentration (0.1% LC1) for 7 days in a culture hood X*vivo* System (BioSpherix, Ltd, USA) (Figure 1A) [18]. Upon 7 days (d7), cells were replaced at atmospheric oxygen concentration for 7 days until day 14. At day 0, 7 and 14, aliquots were analyzed for number cells and apoptosis.

### Lentiviral production, titration, and cell transduction

Lentiviral particles were added to the target cells and incubated for 24 hours. Then, the cells were washed twice in PBS and grown in the presence of medium for 6 days before experimental use. Cells in which targets had been silenced by shRNA (3 different sequences shRNA were tested for all targets) were sorted using GFP or DsRed expression, analyzed by flow cytometry as a homogenous cell population with purity above 98%. To confirm silencing of ATG7, specific protein expression was detected by western-blot. For CD34^+^ cells, infection was performed twice, two and one day before experiment in the presence of polybrene (8 µg/mL). Four days later, an aliquot of cells were analyzed by flow cytometry to measure the percent of infected cells and response to TKI was detected with annexin V-APC. Viability was calculated on both GFP^+^ and GFP^-^ cell population.

### Western Blotting

Protein lysates were prepared according to Gioia *et al* [38]. Protein concentration was measured by the BCA™ Protein Assay (Pierce, Rockford IL, USA) and the lysates were stored at –80°C. Equal amounts of protein were separated by electrophoresis on an SDS-PAGE 12.5 or 15% and transferred to a pvdf membrane as described [39] (Biorad, Marnes-La-Coquette,France). After blocking, the membrane is incubated with primary antibodies and secondary antibodies. Protein–antibody complexes were detected by an enhanced chemiluminescence immunoblotting ECL (Perkin Elmer, Courtaboeuf, France).

### Flow cytometry

Cells (10^5^ cells) were incubated for 10 min in 500 µl of Hepes/NaCl buffer with 2 mM Ca^2+^, 2µl of Annexin V- APC and 0.25 µg of propidium iodine (PI) before flow cytometry analysis on Facs Canto II. At least ten thousand events were acquired for statistical analysis. Detection of apoptosis by annexin V labeling was performed according to the manufacturer instructions (Biolegend). Ki-67 detection was performed on PFA-fixed cells after permeabilization for 5 min using 0.1% Triton X-100. Phycoeryhtrine-conjugated antibody against Ki-67 was incubated for 30 min at 1 µg/ml. After washing, pelleted cells were resuspended in Hepes/NaCl buffer and analyzed by flow cytometry. Phycoeryhtrine-conjugated Isotype was used as control.

### Autophagy inhibition by RNA silencing of ATG7

To inhibit autophagy, HIV-1 lentivirus-based vectors were used to introduce shRNAs against luciferase or ATG7 as already reported [40]. To confirm autophagy inhibition by silencing of ATG7 cells were grown in nutrient deprived medium (HBSS) in the absence or in the presence of Bafilomycin A and both LC3B I and II form were detected by western-blot.

### Autophagy inhibition by PIK-III

PIK-III was dissolved in DMSO at 20 mM. Dose response experiment was performed to determine the concentration which inhibits autophagy with minimal apoptosis using K562 cells expressing the mCherry-GFP-LC3 protein (Kindly provided by Dr Soengas, Madrid, Spain) [41]. K562 cells were incubated with 0, 0.3, 1 and 3 µM of PIK-III and cells were analyzed by flow cytometry after annexin V-APC labelling. PIK-III increases GFP and mCherry fluorescence in a dose dependent manner and 1 µM was chosen as the concentration inhibiting autophagy with no significant apoptosis.

### Statistical analysis

A Wilcoxon test was used to calculate differences between means; differences were considered significant only when *p* ≤ 0.05 or 0.01 as indicated and shown by an asterisk ^*^.

## SUPPLEMENTARY MATERIALS FIGURES


