# Supplementary material for: Chronic myeloid leukemia progenitor cells require autophagy when leaving hypoxia-induced quiescence
Source: Oncotarget. 2017 Jun 30;8(57):96984–92. doi: 10.18632/oncotarget.18904 (PMC5722539; doi:10.18632/oncotarget.18904)
Supplement: Supplementary file 1 [file oncotarget-08-96984-s001.pdf]

Chronic myeloid leukemia progenitor cells require autophagy when leaving hypoxia-induced quiescence

Supplementary Materials

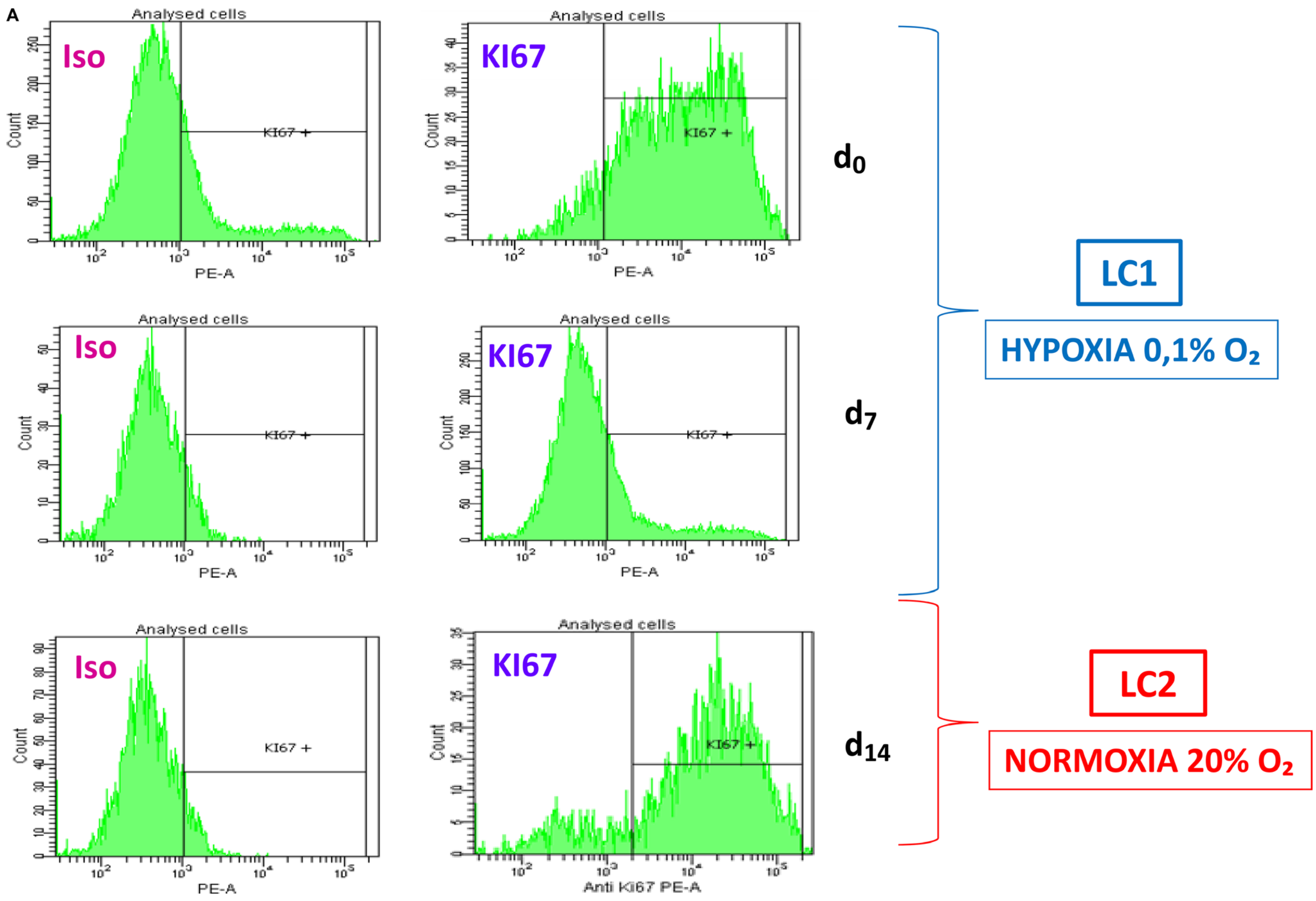

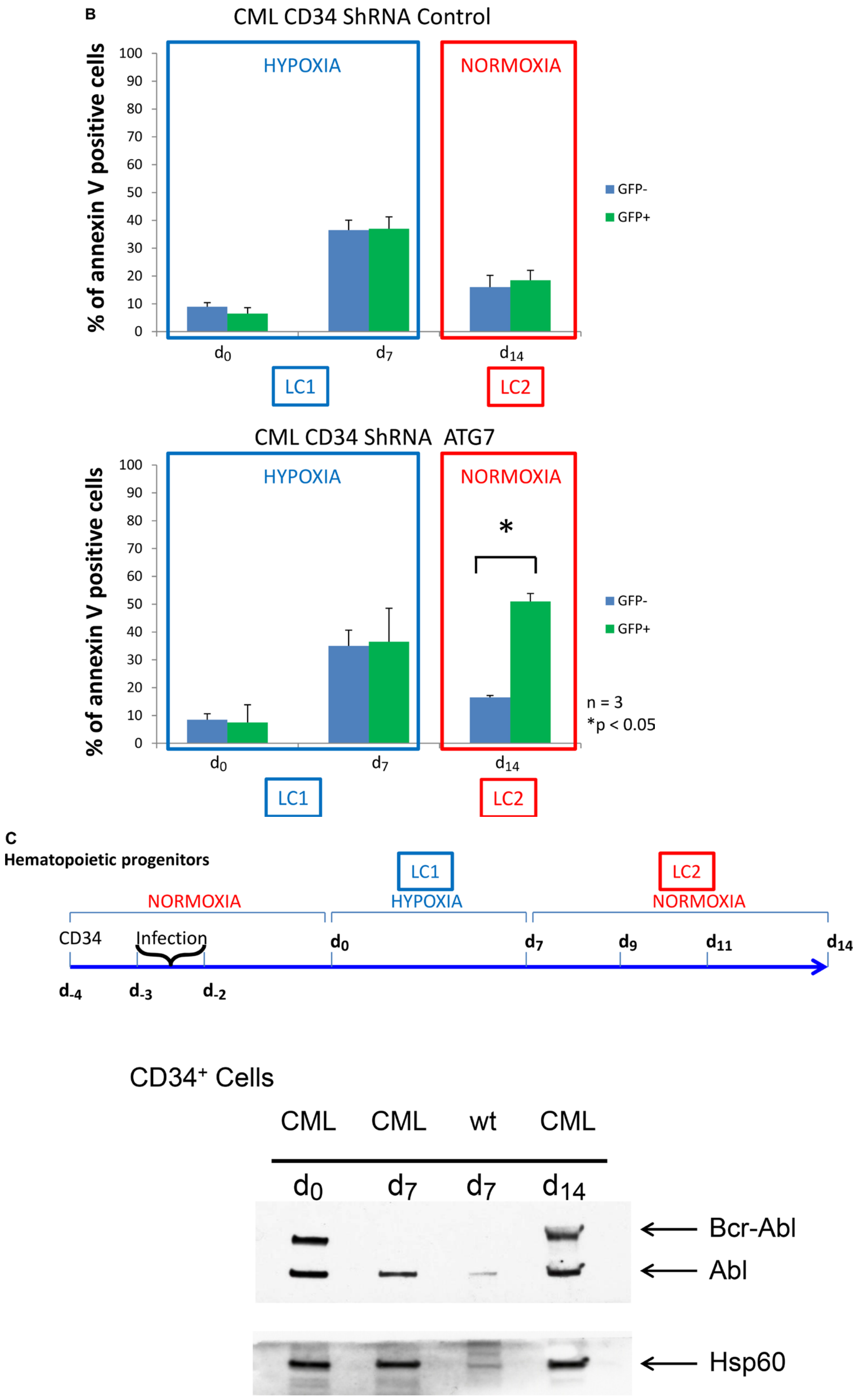

Supplementary Figure 1: (**A**) CML CD34$^+$ cells transduced with a shRNA against ATG7 were cultured at low O_2 concentration (0.1% O_2) for 7 days (LC1). Upon 7 days , cells were replaced at atmospheric O_2 concentration and grown for 7 more days (LC2). At days 0, 7 and 14, aliquot (5.10^5 cells) were fixed in 4% paraformaldehyde for 15 min. Then, permeabized with 0.1% Triton X-100 and then washed in PBS. Phycoerythrine-coupled antibody against Ki67 was incubated for 30 min and analysed by flow cytometry. Results are from one experiment representative of 3. (**B**) CML CD34$^+$ cells were transduced with a shRNA against luciferase (shRNA Control) or a shRNA against ATG7 (ShRNA ATG7). CML CD34$^+$ cells were cultured at low O2 concentration (0.1% O_2) for 7 days (LC1). Upon 7 days, cells were replaced at atmospheric O_2 concentration and grown for 7 more days (LC2). At indicated time, aliquot were analyzed for apoptosis by flow cytometry using annexin V-APC labelling on the two population GFP$^-$ and GFP$^+$. Results are from at least 3 experiments. Significance between GFP- and GFP+ cells was quantitated using Wilcoxon test. (**C**) CML and normal CD34$^+$ cells were cultured at low O_2 concentration (0.1% O_2) for 7 days (LC1). Upon 7 days, cells were replaced at atmospheric O_2 concentration and grown for 7 more days (LC2). At days 0, 7 and 14, aliquot (10^6 cells) were lysed in ripa buffer and Bcr-Abl was detected by western blot. For normal CD34$^+$ cells only the time at day 7 was analyzed.

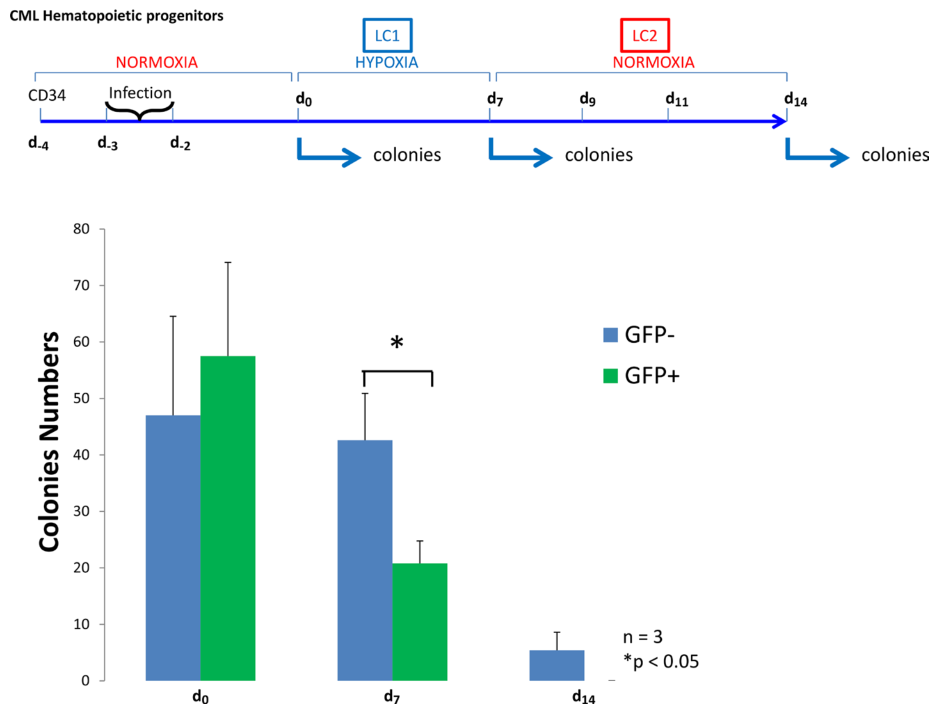

Supplementary Figure 2 : CML CD34$^+$ cells treated as described in Figure 2 were seeded in methyl cellulose at days 0, 7 and 14. Number of GFP$^-$ and GFP$^+$ colonies were counted in triplicates in three separated experiments. Results are expressed as total number of colonies. Significance between GFP$^-$ and GFP$^+$ colonies was quantitated using Wilcoxon test and show by an asterisk when $p < 0.05$.

A

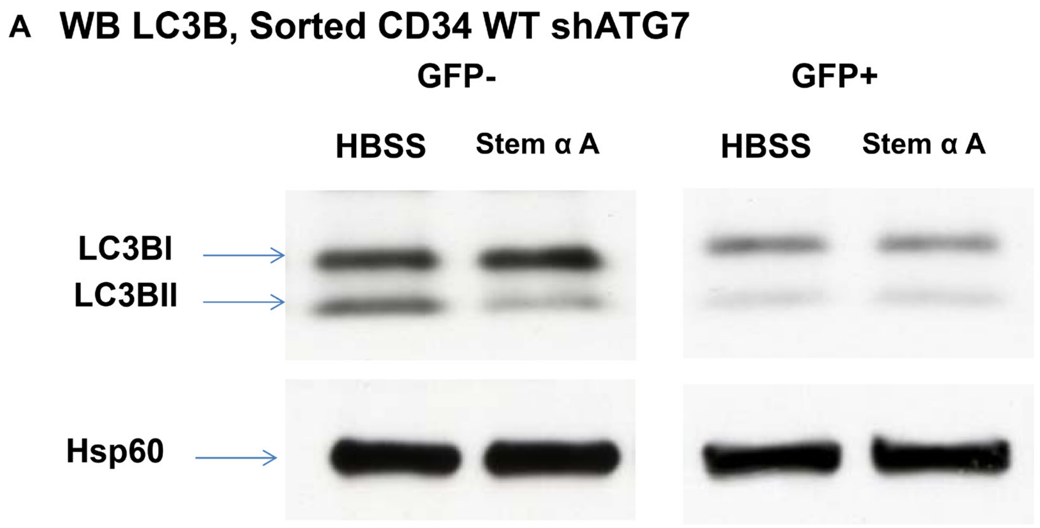
WB LC3B, Sorted CD34 WT shATG7
GFP-
GFP+
HBSS
Stem α A
HBSS
Stem α A
LC3BI
LC3BII
Hsp60

B

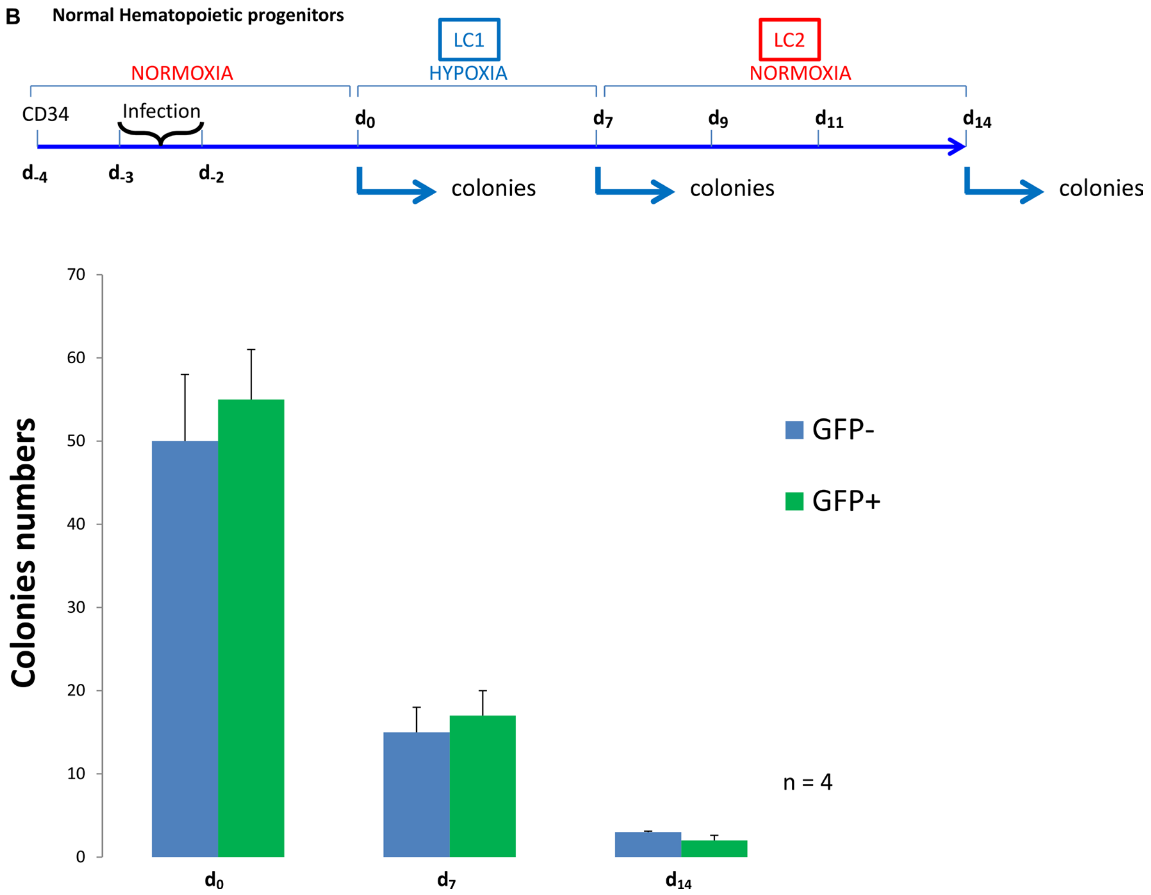
Normal Hematopoietic progenitors
LC1
LC2
NORMOXIA
HYPOXIA
NORMOXIA
CD34
Infection
d0
d7
d9
d11
d14
d-4
d-3
d-2
colonies
colonies
colonies
70
60
50
40
30
20
10
0
Colonies numbers
GFP-
GFP+
n = 4
d0
d7
d14

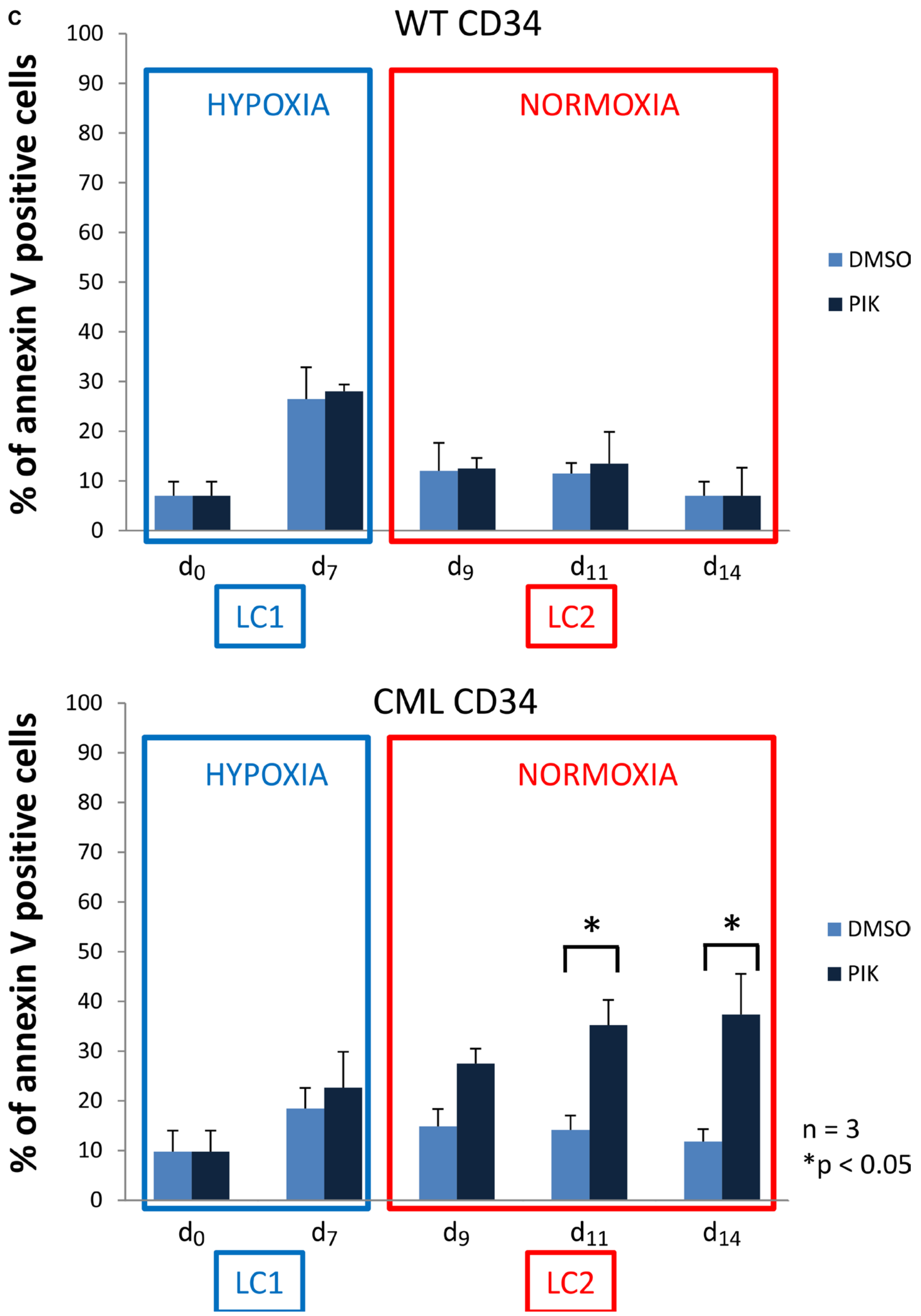

Supplementary Figure 3: (**A**) Normal CD34+ cells were transduced with a shRNA against ATG7. Upon cell sorting, GFP^- and GFP^+ cells were incubated in growing medium (stem a A) or minimal medium (HBSS) for 4 hours. Cells were then lyzed and autophagy operating mechanism was check by detecting the conversion of microtubule associated light chain 3B-I in LC3B-II. As control loading , Hsp60 was detected. (**B**) Normal $CD34^+$ progenitor cells treated as described in Figure 3 were seeded in methyl cellulose at days 0, 7 and 14. Number of GFP^- and GFP^+ colonies were counted in triplicates in 4 separated experiments. Results are expressed as total number of colonies. (**C**) Normal and CML $CD34^+$ cells were cultured at low O_2 concentration (0.1% O_2) for 7 days (LC1). Upon 7 days, cells were replaced at atmospheric O2 concentration and grown in the presence of vehicle alone (DMSO) or 1 µM PIK-III (PIK) for 7 more days (LC2). At indicated time, aliquots were analyzed for apoptosis by flow cytometry using annexin V-APC labelling. Results are from at least 3 experiments. Significant difference between treated and untreated cells was quantitated using Wilcoxon test and show by an asterisk when $p < 0.05$.
